# Single medium-sized hepatocellular carcinoma treated with sequential conventional transarterial chemoembolization (cTACE) and microwave ablation at 4 weeks versus cTACE alone: a propensity score

**DOI:** 10.1186/s12957-022-02643-w

**Published:** 2022-06-10

**Authors:** Zi-yi Zhu, Mu Yuan, Pei-Pei Yang, Bo Xie, Jian-zhu Wei, Zhong-qiang Qin, Zhen Qian, Zhao-Ying Wang, Long-Fei Fan, Jing-yu Qian, Yu-lin Tan

**Affiliations:** Department of Interventional Radiology, The First Affiliated Hospital of Bengbu Medical Colleague, 287 Changhuai Road, Bengshan District, Bengbu, 233004 China

**Keywords:** Microwave ablation, Conventional transarterial chemoembolization, Combined treatment, Hepatocellular carcinoma, Time to progression, Survival

## Abstract

**Background:**

Microwave ablation (MWA) is a potentially curative treatment for unresectable patients with hepatocellular carcinoma (HCC) ≤ 3 cm, while its therapeutic efficacy decreases significantly for HCC > 3cm. Previous studies have demonstrated that conventional transarterial chemoembolization (cTACE) combined with MWA (cTACE-MWA) may improve local tumor control rate and reduce the recurrence rate for HCC > 3cm. However, there have been few study designs to analyze the clinical efficacy of cTACE-MWA for medium-sized HCC (3–5cm). Therefore, this study aims to compare the clinical efficacy and safety of cTACE-MWA with cTACE alone for a single medium-sized HCC of 3–5 cm in diameter.

**Methods:**

We retrospectively investigate the data of 90 patients with a single medium-sized HCC who were referred to our hospital and underwent cTACE-MWA or cTACE alone from December 2017 to March 2020. Then, patients were identified with propensity score-matched (1:1). The local tumor response to treatment and time to progression (TTP) were compared using mRECIST criteria between the cTACE-MWA group and the cTACE group.

**Results:**

A total of 42 patients were included after matching (cTACE-MWA: 21; cTACE: 21). Comparing with cTACE, cTACE-MWA demonstrate significantly better local tumor control (ORR: 95.2% vs 61.9%, *p* = 0.02; DCR: 95.2% vs 66.7%, *p* = 0.045) and TTP (median 19.8 months vs 6.8 months, *p* < 0.001). The 1- and 2-year cumulative probabilities of OS were 100% and 95% in the cTACE-MWA group, which were significantly higher than those in the cTACE group (95% and 76%) (*p* = 0.032). Multivariate Cox regression analysis illustrates that cTACE-MWA was associated with better TTP (hazard ratio, 0.28; 95% CI: 0.1, 0.76; *p* = 0.012), but tumor size was associated with worse TTP (hazard ratio, 1.71; 95% CI: 1.01, 2.89; *p* = 0.045).

**Conclusions:**

cTACE followed by MWA improved TTP and OS in patients with a single medium-sized HCC, and no major complication was observed in this study.

## Background

Hepatocellular carcinoma (HCC) ranked sixth as the most frequent malignancy and fourth as the most prevalent cancer-related death worldwide [1]. According to Barcelona Clinic Liver Cancer (BCLC) stage recommendation, for single medium-sized HCC (range 3–5cm), surgical resection (SR) and liver transplantation (LT) remain the first-line treatment with improved medium- and long-term survival. However, only a few patients are suited for these curative treatments due to liver donor deficiency or comorbidity of cirrhosis [2–5]. Radiofrequency ablation (RFA) is another potentially curative therapy strategy recommended by the BCLC guideline for patients with HCC ≤ 3 cm, which can provide a comparable survival benefit to SR [6–9]. For HCC > 3 cm, the therapeutic efficacy of RFA decreases significantly with increasing tumor size [10, 11]. Therefore, the guideline still did not explicitly recommend RFA as a first-line treatment for HCC > 3 cm.

Microwave ablation (MWA) has emerged as an effective alternative method to RFA for larger tumors [12]. Although there are controversies about whether MWA is superior to RFA, MWA exhibit several theoretical advantages, such as larger ablation zones can be created in a short time and are less susceptible to “heat sink” [13, 14]. In addition, the synchronous multi-antenna approach has been used in clinical practice and has shown the ability to eradicate larger HCC [15].

TACE worked by selectively delivering chemotherapy agents and cutoff the feeding artery has been firmly established as a critical option for BCLC stage B HCC. TACE followed by local ablation may create a larger ablation zone, improving the coverage of undetected micro-metastasis and chemotherapy agent intake, and thus, reducing the possibility of recurrence [16]. The previous study has validated cTACE combined with RFA as an effective treatment for medium-sized HCCs, but the study on cTACE combined with MWA is still scarce [17–19].

To focus on the study purpose, only patients with a single HCC range 3–5cm in size were included. Moreover, a propensity score matching (PSM) analysis was performed to reduce the impact of confounding bias at baseline. The purpose of the present study aims to compare the efficacy and safety of cTACE-MWA combination treatment versus cTACE monotherapy for the medium-sized HCC and analyze the predictors that might influence the superiority of one treatment over the other.

## Methods

### Study design

This retrospective study was approved by the institutional review board of the first affiliated hospital of Bengbu medical colleague and complied with the Declaration of Helsinki. A waiver of informed consent was granted. We retrospectively investigated the clinical data of consecutive patients who received cTACE-MWA or cTACE therapy from December 2017 to March 2020. The diagnosis of HCC was determined using the guidelines of the American Association for liver diseases, and an ultrasound-guided liver biopsy was performed if the diagnosis is unclear [20]. All patients undergoing cTACE or cTACE-MWA were ineligible or refuse to SR or LT after discussion by a multidisciplinary local tumor board that included hepatologists, radiologists, oncologists, and anesthesiologists. Patients underwent cTACE or cTACE-MWA therapies based on the patient’s decision after being informed about the advantages and disadvantages of the two treatments respectively, such as treatment outcomes, morbidities, and costs.

### Patient selection criteria

**Fig. 1:**
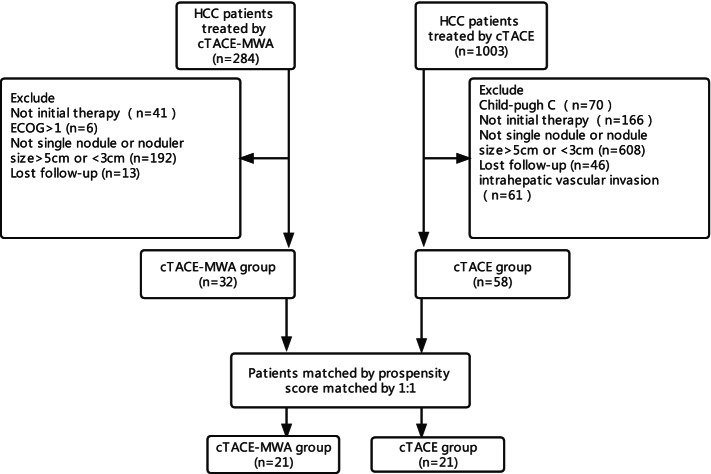
Flowchart of patient selection

Patients were selected according to the following criteria: (1) tumor measuring 3–5cm, (2) single nodule, (3) Child-Pugh classification A or B, and (4) Eastern Cooperative Oncology Group (ECOG) performance status of 0 or 1, and excluded according to the following criteria: (1) BCLC stage C and (2) miss data (Fig. 1).

### cTACE

The transarterial chemoembolization procedure was performed under conscious sedation in an interventional suite by board-certified interventional radiologists in our center. Under local anesthesia using 5% lidocaine, the puncture of the common femoral artery was performed using the Selinger technique, after which a 5F RH catheter (Terumo, Tokyo, Japan) was introduced with a combination of the 0.035-inch hydrophilic guidewire (Terumo, Tokyo, Japan) to catheterize the celiac, superior mesenteric artery, and any suspected artery feeding the tumor. Digital subtraction angiography was performed to evaluate tumor location and size. The distal target artery was super selectively catheterized with a microcatheter (Terumo, Tokyo, Japan). Then, chemoembolization was performed using an emulsion of epirubicin (20–40mg; Pharmorubicin; Pfizer, Wuxi, China) in the iodized oil (1–10ml; Lipiodol Ultra-Fluide; Hengrui, Jiangsu, China), depending on liver function, tumor size, and vascular supply. Further embolization with gelatin sponge particles (Hangzhou Alc, Hangzhou, China) was finally performed until arterial flow stasis and no tumor staining after repeat angiography.

### cTACE-MWA combination

The MWA procedure was performed 4 weeks after the cTACE procedure with a clinical MWA system at a 2450-MHz frequency (KY-2000, Canyou Medical Inc, Jiangsu, China). The MWA system can simultaneously drive two independent 15-gage (1.9cm) water-cooled antennas with a power output range of 1 to 100 W to deliver microwave energy into tumor tissue. All procedures were performed in our institution and percutaneously under CT guidance by two board-certified operators with experience of more than 5 years. After conscious sedation and local anesthesia, two antennas were inserted simultaneously with a separation distance of 1.5–2cm in the upper part of the tumor with outpower set at 50–60 W for 5–10 min in a session. Then, the antennas were slowly removed, and reinserted in the lower part of the tumor, repeating the same protocol. The outpower setting, ablation time, and placement of antennas were depended on the tumor size, shape, and locations. After tumor ablation, track ablation was performed for all patients to avoid tumor bleeding and seeding.

### Follow-up

The primary outcome was TTP and local tumor response. TTP was defined as the interval between the first administration of study treatment and tumor progression, death, or the end of the study (Nov 2021). Local tumor response was evaluated at the first month and 6 months after treatment by objective response rate (ORR) and disease control rate (DCR) and progressive disease (PD). According to the modified Response Evaluation Criteria in Solid Tumors (mRECIST), ORR was defined as complete response (CR) rate plus partial response (PR) rate, and DCR was defined as ORR plus stable disease (SD) rate. The secondary outcome was overall survival (OS), OS was calculated from the first treatment to death or the end of the study (Nov 2021). Enhanced liver CT/MRI scan and laboratory tests were performed to evaluate tumor response at 1 and 3 months after treatment and thereafter at 3-month intervals during the first years, then at approximately 6-month intervals thereafter. All follow-up images were reviewed by consensus by two board-certified radiologists in our institution (each with more than 5 years of experience in abdominal radiology and liver thermal ablation). Treatment-related complications were assessed by follow-up scan based on the Society of Interventional Radiology (SIR) grading system [21].

### Statistical analysis

We used the statistical software packages R (http://www.R-project.org, The R Foundation) and Free Statistics software version 1.2 for all analyses, and a two-sided *p* < 0.05 was considered statistically significant. Categorical variables were expressed as proportions (%). Continuous data were expressed as mean ± standard deviation (SD) or median and interquartile range (IQR), as appropriate. To minimize the potential bias, we performed a propensity score matching (PSM) using a 1:1 nearest neighbor matching algorithm with a 0.2 caliper width. The variables selected to generate the propensity score included age, sex, HBV, HCV, AFP, CP, tumor size, portal hypertension, platelet, TBIL, CRE, and INR. Multivariable Cox regression analyses were adopted to assess the independent association between prognostic factors and TTP rate. Survival curves were plotted by Kaplan-Meier and log-rank analyses.

## Result

### Baseline characteristics

Between June 2017 and July 2019, out of a total of 1287 patients with HCC who received either cTACE or cTACE+MWA identified, 1197 patients were excluded according to the exclusion criteria. Finally, the remaining 90 patients met the inclusion criteria.

The baseline characteristics of all patients are listed in Table 1. Before PSM, patients in the cTACE-MWA group had a larger average tumor diameter compared with those in the cTACE group (3.3 vs 4.0, *p* = 0.009). After PSM, 21 patients in the cTACE-MWA group were successfully matched by applying 1:1 with an equal number of patients in the cTACE group, and no significant differences were observed between the two groups (Table 2).

**Table 1:** Baseline characteristics of the study participants (before PSM)

Variables	Total (*n* = 90)	Unmatched cohort		*p*
		cTACE (*n* = 58)	cTACE+MWA (*n* = 32)	
Age, mean ± SD	57.9 ± 10.4	57.8 ± 10.6	58.1 ± 10.2	0.886
Sex, *n* (%)				0.74
Female	11 (12.2)	8 (13.8)	3 (9.4)	
Male	79 (87.8)	50 (86.2)	29 (90.6)	
HBV, *n* (%)				0.089
No	22 (24.4)	18 (31)	4 (12.5)	
Yes	68 (75.6)	40 (69)	28 (87.5)	
HCV, *n* (%)				0.343
No	85 (94.4)	56 (96.6)	29 (90.6)	
Yes	5 ( 5.6)	2 (3.4)	3 (9.4)	
AFP, *n* (%)				0.361
<200ng/ml	63 (70.0)	43 (74.1)	20 (62.5)	
≥200ng/ml	27 (30.0)	15 (25.9)	12 (37.5)	
CP, *n* (%)				1
A	81 (90.0)	52 (89.7)	29 (90.6)	
B	9 (10.0)	6 (10.3)	3 (9.4)	
Tumor size, median (IQR)	3.4 (3.0, 4.3)	3.3 (3.0, 4.0)	4.0 (3.2, 4.5)	0.009
Portal hypertension, *n* (%)				0.157
No	43 (47.8)	24 (41.4)	19 (59.4)	
Yes	47 (52.2)	34 (58.6)	13 (40.6)	
PLT (×10^9^/L), median	99.0 (69.2, 149.8)	86.5 (67.2, 145.5)	113.5 (81.2, 151.5)	0.228
TBIL (μmol/L), median (IQR)	14.2 (9.1, 18.1)	14.5 (9.4, 19.0)	12.2 (8.3, 16.7)	0.1
CRE (μmol/L), median (IQR)	65.0 (59.0, 70.0)	65.0 (59.2, 70.8)	64.0 (59.0, 68.5)	0.723
INR, median (IQR)	1.1 (1.1, 1.2)	1.1 (1.1, 1.2)	1.1 (1.0, 1.1)	0.033

Notes: data presented are mean±SD, median (Q1–Q3), and *N* (%)

*Abbreviations*: *HBV* hepatic B virus, *HCV* hepatic C virus, *AFP* alpha-fetoprotein, *CP* Child-Pugh Classification, *PLT* platelet, *TBIL* total bilirubin, *CRE* creatinine, *INR* prothrombin time-international normalized ratio

**Table 2:** Baseline characteristics of the study participants (after PSM)

Variables	Total (*n* = 42)	Matched cohort		
		cTACE (*n* = 21)	cTACE+MWA (*n* = 21)	*p*
Age, mean ± SD	59.0 ± 9.4	60.7 ± 9.9	57.3 ± 8.8	0.249
Sex, *n* (%)				1
Female	3 (7.1)	2 (9.5)	1 (4.8)	
Male	39 (92.9)	19 (90.5)	20 (95.2)	
HBV, *n* (%)				1
No	5 (11.9)	3 (14.3)	2 (9.5)	
Yes	37 (88.1)	18 (85.7)	19 (90.5)	
HCV, *n* (%)				1
No	39 (92.9)	19 (90.5)	20 (95.2)	
Yes	3 (7.1)	2 (9.5)	1 (4.8)	
AFP, *n* (%)				1
<200ng/ml	27 (64.3)	14 (66.7)	13 (61.9)	
≥200ng/ml	15 (35.7)	7 (33.3)	8 (38.1)	
CP, *n* (%)				1
A	39 (92.9)	19 (90.5)	20 (95.2)	
B	3 (7.1)	2 (9.5)	1 (4.8)	
Tumor size, median (IQR)	3.4 (3.1, 4.1)	3.4 (3.1, 4.1)	3.5 (3.2, 4.1)	0.612
Portal hypertension, *n* (%)				1
No	19 (45.2)	9 (42.9)	10 (47.6)	
Yes	23 (54.8)	12 (57.1)	11 (52.4)	
PLT (×10^9^/L), median	91.0 (67.2, 138.5)	88.0 (67.0, 137.0)	98.0 (70.0, 139.0)	0.772
TBIL (μmol/L), mean ± SD	13.3 ± 5.7	13.9 ± 6.4	12.7 ± 5.0	0.512
CRE (μmol/L), mean ± SD	66.6 ± 8.9	65.1 ± 8.6	68.0 ± 9.2	0.296
INR, mean ± SD	1.1 ± 0.1	1.1 ± 0.1	1.1 ± 0.1	0.653

*Notes*: data presented are mean ± SD, median (Q1–Q3), and *N* (%)

*Abbreviations*: *HBV* hepatic B virus, *HCV* hepatic C virus, *AFP* alpha-fetoprotein, *CP* Child-Pugh Classification, *PLT* platelet, *TBIL* total bilirubin, *CRE* creatinine, *INR* prothrombin time-international normalized ratio

### Tumor response

We compared the tumor response according to mRECIST criteria between the two groups at the first (M1) and sixth (M6) months after treatment. In the first month, technical success achieved 100% in both groups. However, at M6 after treatment, the cTACE-MWA group had a significantly higher objective response rate (ORR) and disease control rate (DCR) than the cTACE group (ORR: 93.8% vs 56.9%, *p* = 0.001; DCR: 93.8% vs 62.1%, *p* = 0.003).

After PSM, at M6 after treatment, there was still a significant difference of tumor response between the two groups (ORR: 95.2% vs 61.9%, *p* = 0.02; DCR: 95.2% vs 66.7%, *p* = 0.045) (Table 3).

**Table 3:** Local tumor response

	Before PSM, *n* (%)		cTACE+MWA	*p*	After PSM, *n* (%)		cTACE+MWA	*p*
	Total	cTACE			Total	cTACE		
1 month								
Patient, *n*	90	58	32		42	21	21	
CR	62 (68.9)	32 (55.2)	30 (93.8)	0.001	33 (78.6)	13 (61.9)	20 (95.2)	0.02
PR	13 (14.4)	12 (20.7)	1 (3.1)	0.028	2 (4.8)	1 (4.8)	1 (4.8)	1
SD	10 (11.1)	9 (15.5)	1 (3.1)	0.09	5 (11.9)	5 (23.8)	0 (0)	0.048
PD	5 (5.6)	5 (8.6)	0 (0)	0.156	2 (4.8)	2 (9.5)	0 (0)	0.488
ORR	75 (83.3)	44 (75.9)	31 (96.9)	0.152	35 (83.3)	14 (66.7)	21 (100)	0.009
DCR	85 (94.4)	53 (91.4)	32 (100)	0.416	40 (95.2)	19 (90.5)	21 (100)	0.488
6 months								
CR	58 (64.4)	31 (53.4)	27 (84.4)	0.007	31 (73.8)	12 (57.1)	19 (90.5)	0.035
PR	5 (5.6)	2 (3.4)	3 (9.4)	0.343	2 (4.8)	1 (4.8)	1 (4.8)	1
SD	3 (3.3)	3 (5.2)	0 (0)	0.55	1 (2.4)	1 (4.8)	0 (0)	1
PD	24 (26.7)	22 (37.9)	2 (6.2)	0.003	8 (19.0)	7 (33.3)	1 (4.8)	0.045
ORR	63 (70.0)	33 (56.9)	30 (93.8)	0.001	33 (78.6)	13 (61.9)	20 (95.2)	0.02
DCR	66 (73.3)	36 (62.1)	30 (93.8)	0.003	34 (81.0)	14 (66.7)	20 (95.2)	0.045

*Abbreviations*: *CR* complete response, *PR* partial response, *SD* stable disease, *PD* progressive disease, *ORR* complete response + partial response, *DCR* ORR + stable disease

### Tumor progression and association of risk factors

**Fig. 2:**
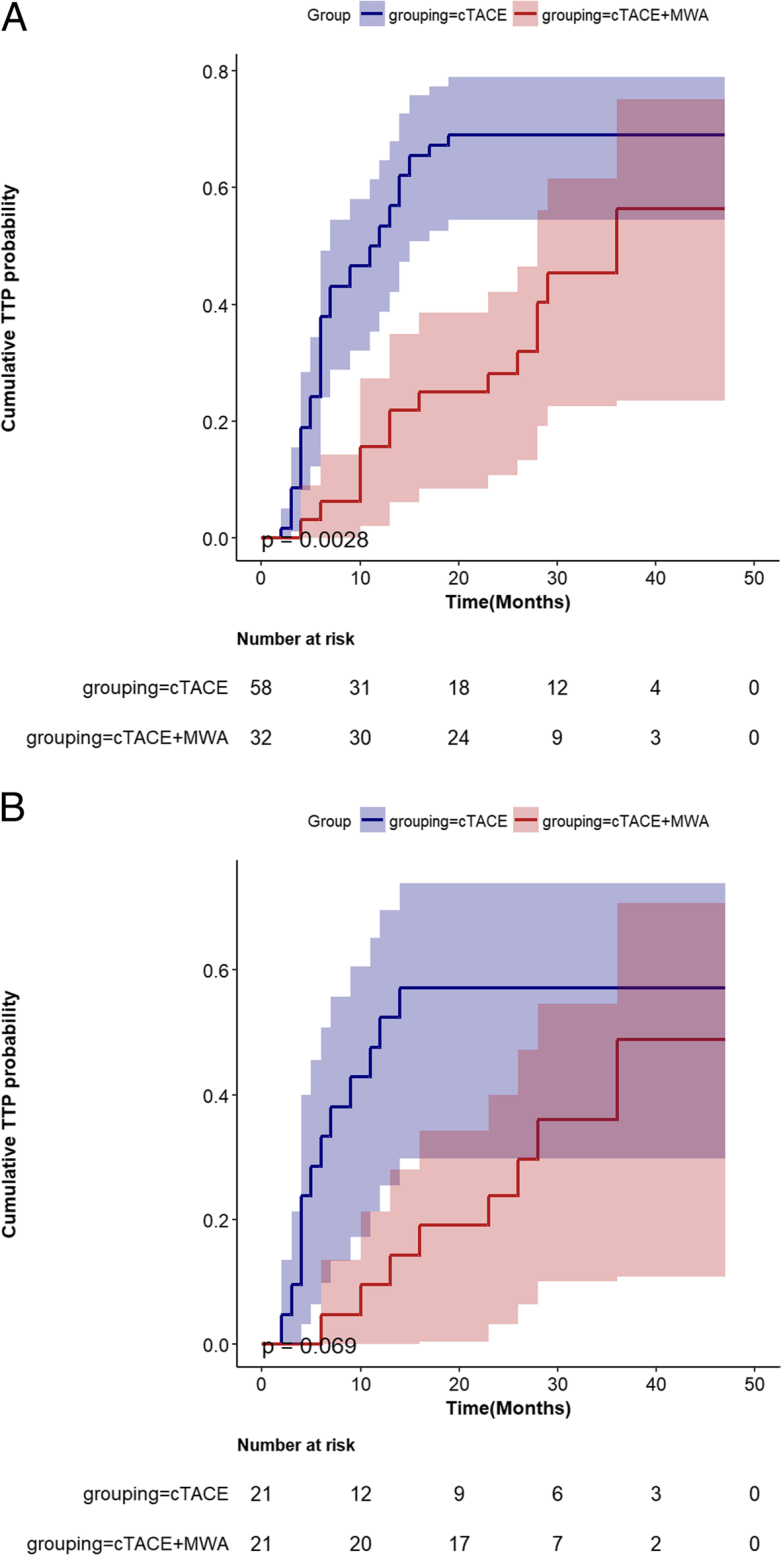
Cumulative time to progression (TTP) rate curves for patients who underwent conventional transarterial chemoembolization (cTACE) or cTACE combined with microwave ablation (cTACE-MWA) before (**A**) and after (**B**) propensity score matching

For the entire cohort, 14 of 32 (44%) patients in the cTACE-MWA group and 40 of 58 (69%) patients in the cTACE group suffer tumor progression. The cumulative tumor progression rates at 0.5, 1, and 2 years were 6%, 16%, and 28% for the cTACE-MWA group, while 38%, 53%, 69% for the cTACE group (*p* = 0.0028) (Fig. 2A).

After PSM, 8 of 21 (38%) patients in the cTACE-MWA group and 12 of 21 (57%) patients in the cTACE group experienced the disease progression. Moreover, consistent with the result before PSM, the cumulative tumor progression rates at 0.5, 1, and 2 were also better in the cTACE-MWA group than in the cTACE group (4%, 10%, and 24% vs 33%, 38%, and 57%, *p* = 0.069) (Fig. 2B).

As Table 4 shows, in multivariate Cox regression analysis, cTACE-MWA was independently associated with better TTP (hazard ratio, 0.28; 95% CI: 0.1, 0.76; *p* = 0.012), while tumor size was identified as an independent predictor for poor TTP (hazard ratio, 1.71; 95% CI: 1.01, 2.89; *p* = 0.045).

**Table 4:** Multivariate analysis of tumor progression using the Cox regression model

Variable	*p*	HR	95% CI for HR	
			Lower	Upper
Intervention	0.012	0.28	0.1	0.76
Age	0.109	0.97	0.94	1.01
Sex	0.071	0.42	0.16	1.08
HBV	0.187	0.59	0.26	1.3
HCV	0.107	0.17	0.02	1.46
AFP	0.398	1.42	0.63	3.21
CP	0.26	1.97	0.61	6.37
Tumor size	0.045	1.71	1.01	2.89
Portal hypertension	0.149	2.33	0.74	7.36
PLT (×10^9^/L)	0.785	1	0.99	1.01
TBIL	0.804	0.99	0.93	1.05
CRE	0.215	1.02	0.99	1.06
INR	0.578	0.32	0.01	17.02

*Abbreviations*: *HBV* hepatic B virus, *HCV* hepatic C virus, *AFP* alpha-fetoprotein, *CP* Child-Pugh Classification, *PLT* platelet, *TBIL* total bilirubin, *CRE* creatinine, *INR* prothrombin time-international normalized ratio

For the entire cohort, tumors treated with cTACE+MWA achieved longer TTP when compared to cTACE alone (median 18 months vs 8 months, *p* <0.001). After PSM, the median time to TTP was 19.8 months for the cTACE+MWA group compared with 6.8 months for the cTACE group (*p* < 0.001).

### Overall survival

**Fig. 3:**
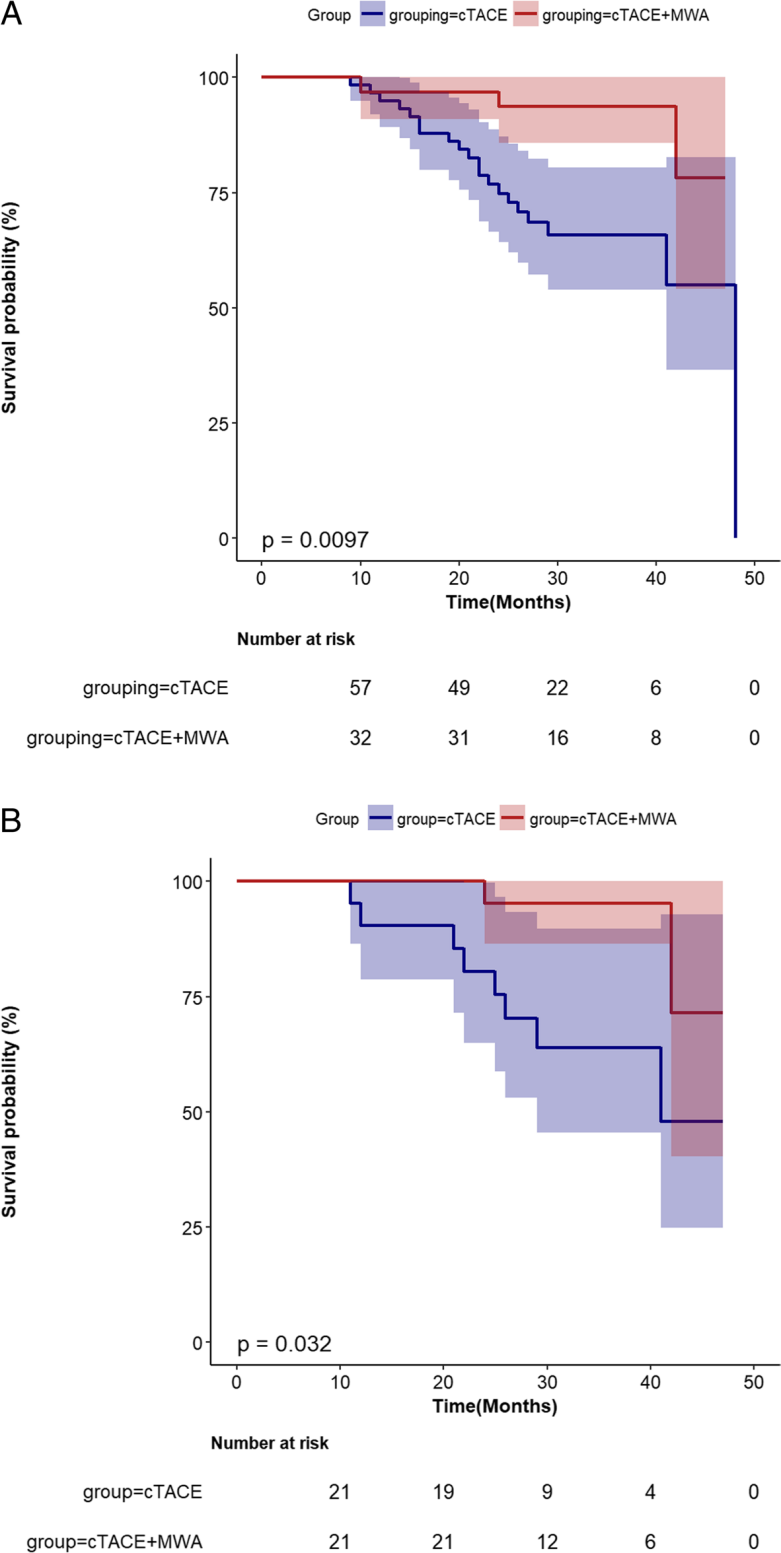
Cumulative overall survival (OS) rate curves for patients who underwent conventional transarterial chemoembolization (cTACE) or cTACE combined with microwave ablation (cTACE-MWA) before (**A**) and after (**B**) propensity score matching

For the entire cohort, the median follow-up duration was 31.5 ± 8.4 months (range 10–47 months) in the cTACE-MWA group and 27.2 ± 8.8 months (range 9–47 months) in the cTACE group (*p* = 0.026). At the end of the follow-up, 3 of 32 (9%) patients in the cTACE-MWA group and 20 of 58 (34%) patients in the cTACE group had died. The 1- and 2-year cumulative probabilities of OS were 97% and 94% in the cTACE-MWA group and 95% and 76% in the cTACE group (*p* = 0.0097) (Fig. 3A).

After PSM, the median follow-up duration was 32.7 ± 7.5 months (range 22–47 months) in the cTACE-MWA group and 29.4 ± 9.9 months (range 12-47 months) in the cTACE group (*p* = 0.233). Two (8%) patients in the cTACE-MWA group and 7 (29%) patients in the cTACE group had died. The 1- and 2-year cumulative probabilities of OS were 100% and 95% in the cTACE-MWA group and 95% and 76% in the cTACE group (*p* = 0.032) (Fig. 3B).

For the entire cohort, 11 of 32 (34%) patients in the cTACE-MWA group developed intra-hepatic recurrence. Of these 11 cases, local tumor recurrence and intra-hepatic new lesions occurred in 4 and 7 patients and were treated with secondary ablation. Additionally, 3 patients suffered from tumor metastasis, including lung, bone, abdominal wall, and retroperitoneal lymph node and managed with systemic therapy. In the cTACE group, 40 of 58 (69%) patients experienced tumor progression, and treatment for tumor progression was as follows: recTACE (*n* = 21), systemic treatment with sorafenib or lenvatinib (*n* = 13). The remaining patients received supportive care therapy because of other concurrent medical comorbidities.

### Complication

No treatment-related mortality was observed in both groups. In the cTACE group, one patient (2%) experienced major complications related to the treatment: liver abscess. In the cTACE-MWA group, 2 of 32 (6%) patients experienced major complications related to the treatment including pneumothorax (*n* = 2). Patients with liver abscess significantly improved after ultrasound-guided percutaneous drainage and anti-inflammatory treatments. Patients with pneumothorax were minor and did not need chest tube placement. All of these complications were non-fatal and cured following treatment. Minor complications (vomiting, fever, nausea, puncture point, or right shoulder pain) were observed in 13 of 32 patients (41%) in the cTACE-MWA group compared to 38 of 58 patients (66%) in the cTACE group. All of these minor complications were transient and relieved before discharge.

## Discussion

The present study demonstrated that cTACE combined with MWA is effective and safe for solitary medium-sized HCC. The TTP of the cTACE-MWA combination therapy group was significantly lower compared with that noted in the cTACE monotherapy group. Compared with the cTACE group, the cTACE-MWA group did not increase the incidence of postoperative complications.

The use of cTACE or RFA as a treatment option for patients with HCC > 3cm has been previously studied while these treatments have been associated with a high risk of local and distant tumor recurrence at 5 years [11, 18, 22, 23]. Compared with cTACE, drug-eluting microsphere (DEB-TACE) is characterized by loading chemotherapy on beads and releasing it over time, which has been widely applied for the transvascular treatment of various tumors. Nevertheless, response rates and survival are not different between the technique in patients with HCC [24, 25]. Several retrospective and randomized studies demonstrated that a combination of cTACE and RFA in HCC 3–5cm increased local control compared to cTACE or RFA alone [17–19]. However, there is very little data regarding the use of MWA in combination with cTACE for patients with medium-sized HCC.

As a promising new thermal technique, MWA was in large part to overcome the shortcomings of RFA, such as less being affected by heat sink effect, larger ablation zone, and more predictable ablations [13, 14]. Moreover, several thermo-protective techniques and simultaneous multi-antenna ablation have been developed to increase ablation volume while limiting the risks of complications [26]. In a subgroup analysis, Chen et al. show a better TTP in the cTACE-MWA group compared with the cTACE group for patients with HCC 3–5cm. Smolock et al. subsequently reported 3–5-cm tumors treated with cTACE-MWA improve local tumor control and contribute to prolonged tumor recurrence compared with cTACE monotherapy, although the statistical significance was not reached (complete response rate: 65% vs 38%, *p* = 0.11; LTP rate: 34.8% vs 62.5%, *p* = 0.11) [27]. In a recent RCT trial, Zaitoun et al. demonstrated that the mean progression-free survival (PFS) was significantly higher in the cTACE-MWA group than in the cTACE and MWA group (PFS: 22.3 months vs 15.4 months vs 16.7 months, *p* < 0.001) [28]. In the present study, the cumulative tumor progression rate in the cTACE-MWA combination group was significantly lower than that in the cTACE group (*p* < 0.01), and TTP was longer in the cTACE-MWA group than in the cTACE group. Additionally, multivariate Cox regression analysis showed cTACE-MWA combination treatment was an independent factor against tumor progression.

In this study, the use of cTACE-MWA combination therapy achieves an improved local tumor control compared with cTACE alone. After receiving combination therapy, 90.5% of patients achieved ORR compared with 57.1% of patients who received cTACE alone (*p* = 0.035). Moreover, the DCR in the cTACE-MWA group was also significantly higher than those in the cTACE group (*p* = 0.045). The increased local tumor control may be due to the synergistic effect of two mobilities: (1) cTACE was performed 2–4 weeks before ablation, which could decrease the vascularity of the treated area and enlarge the ablation zone. (2) The range of tumor and underlying satellite nodules are labeled before MWA [29]. Thus, it provides guidance and decreases the chance of tumor recurrence. Our findings are close to those of prior studies.

Notwithstanding the timing of the sequential treatment is still debated, cTACE followed by ablation are mostly common treatment algorithms [17, 18, 22, 23, 27–30]. Some authors considered the shorter interval between the two interventions potentially enhancing the therapeutical efficacy by reducing the cooling effect of hepatic blood perfusion using embolization [30, 31]. Yuan et al. [32] reported combined cTACE and RFA of large HCC (mean 9 cm, range 5.3–17.9 cm) performed simultaneously under the cone-beam CT (CBCT) or Angio-CT guidance and achieved promised oncologic results (the mean PFS and OS time were 14 months and 18 months for the Angio-CT group; 13 months and 17 months for the CBCT group). In a similar study, Wang et al. [33] reported a desirable therapeutic effect on local tumor control (CR and CR+PR were 90.4% and 100% at the 1-month follow-up). However, the theoretical basis of most studies is based on RFA for large HCC (>5 cm) and there are no separate prospective studies to verify the superiority of shorter treatment intervals. At our institution, patients usually undergo MWA 4 weeks after cTACE, and the reasons are as follows: (1) the prolonged treatment interval may facilitate recovery of liver function and relief of post-embolization syndromes. (2) The iodized oil retained in hepatic parenchyma was washed out by hepatic blood flow to evaluate the tumor margin accurately. Therefore, further studies are necessary to be performed to verify a reasonable interval between the two interventions.

Recently, several retrospective studies have demonstrated that patients with early- and intermediate-stage HCC treated with balloon-occluded TACE (B-TACE) achieve better tumor response and prolonged overall survival than those who undergo cTACE treatment [34–38]. The B-TACE procedure is characterized by using a balloon microcatheter inflated within the tumor-feeding arteries, redistributing the blood flow towards a low resistance area such as a hypervascular HCC, which leads to the dense lipiodol accumulation. Moreover, the temporary occlusion of the tumor feeding artery potentially modifies the water content of the occluded vascular segment, thereby enlarging the ablation volume. In a retrospective multicenter study, Lucatelli et al. [38] reported patients with liver malignancies > 3cm treated with B-TACE followed by ballon-occluded MWA (B-MWA) achieve a much larger necrotic area than that a single antenna could be created. In addition, a CR rate of 85.7% and an OR rate of 95.3 at 6 months are similar to those of the present study (CR: 90.5%; OR: 90.5%). It is worthy to note that a single antenna simultaneously combined with B-TACE can result in a therapeutic outcome equivalent to the present study with multiple antennas synergistic ablation, and even less cost for patients. However, whether the combination of B-MWA with B-TACE could break down the barrier of 5 cm for curative ablation needs to be confirmed by prospective randomized trials.

Ultrasound and computer tomography (CT) are the two most commonly used guidance modalities. For inconspicuous and small lesions, contrast-enhanced ultrasound guidance has been shown to reduce the risk of LTP owing to its real-time ability as compared to computed tomography guidance [39]. However, there are some limitations for ultrasound-guided ablation for medium or large tumors: safe and precise percutaneous targeting often challenges for most operators using ultrasound guidance when simultaneous placement of multiple ablation antennas; the subphrenic tumor may be inadequately scanned due to the overlapping lung and ribs; microbubbles within and around the tumor, formulating during the ablation procedure, may occlude ultrasound beam and prevent repositioning of the antennas. Unlike ultrasound, CT images are unaffected by the lung and rib [40]. On the other hand, the accumulation of iodized oil in a tumor after cTACE is helpful for targeting and precise placement of multiple antennas.

In terms of complications, no significant differences in major complications were observed between the cTACE-MWA group and the cTACE group. In the cTACE-MWA group, the major complication was a pneumothorax, which was observed in 33% of subdiaphragmatic tumors treated with a transpleural approach. In comparison, previous literature reported pneumothorax rate of transpleural MWA/RFA range between 35.3 and 92.9%, which is significantly higher [41–43]. Moreover, two cases were only small pneumothorax and no need for further intervention or hospitalization. In addition, the minor complications (including vomiting, fever, nausea, puncture point, or right shoulder pain) were slightly prevalent in the cTACE-MWA group compared with the cTACE group, while the complications were transient and relieved before discharge. Smolock et al. [27] reported no major complications were observed in either the cTACE-MWA group or the cTACE group for 3–5cm HCC. Mohamed et al. [28] demonstrate that minor complications were commonly observed in the cTACE-MWA group, which was similar to our study. The main reason may be attributed to the combination treatment introducing additional risk with each procedure. In conclusion, our study also suggested that both cTACE-MWA and MWA were safe treatments for medium-sized HCC.

Our study has several noteworthy limitations. First, it was a retrospective nonrandomized study. Some degree of selection bias was inevitable, despite propensity score matching analysis was applied to adjust this potential confounder. Second, the two treatment groups were not a randomized assignment, although the baseline variables were not significantly different after propensity matching, patients underwent MWA whose tumor was located in a position where the antennas could be inserted and held safely, and refractory to cTACE treatment. Third, the number of patients enrolled was relatively small and conducted at a single center; thus, a large-scale, prospective study should be conducted.

## Conclusions

In conclusion, this study demonstrates that conventional cTACE followed by MWA is a safe and effective treatment for patients with solitary 3–5-cm HCC compared with cTACE monotherapy.

## Data Availability

The datasets used and/or analyzed during the current study are available from the corresponding author upon reasonable request.

## Abbreviations

MWA: Microwave ablation
HCC: Hepatocellular carcinoma
cTACE: Conventional transarterial chemoembolization
cTACE-MWA: Conventional transarterial chemoembolization combined with MWA
TTP: Time to progression
ORR: Objective response rate
DCR: Disease control rate
OS: Overall survival
